# Influence of Blanching on the Gene Expression Profile of Phenylpropanoid, Flavonoid and Vitamin Biosynthesis, and Their Accumulation in *Oenanthe javanica*

**DOI:** 10.3390/antiox11030470

**Published:** 2022-02-26

**Authors:** Sunjeet Kumar, Xinfang Huang, Qun Ji, Abdul Qayyum, Kai Zhou, Weidong Ke, Honglian Zhu, Guopeng Zhu

**Affiliations:** 1 Key Laboratory for Quality Regulation of Tropical Horticultural Crops of Hainan Province, School of Horticulture, Hainan University, Haikou 570228, China; 184224@hanainu.edu.cn; 2 Institute of Vegetables, Wuhan Academy of Agricultural Sciences, Wuhan 430207, China; huangxinfang99@163.com (X.H.); jiqun741@sina.com (Q.J.); 13607157304@163.com (K.Z.); wdke63@163.com (W.K.); 3 Department of Agronomy, The University of Haripur, Haripur 22620, Pakistan; aqayyum@uoh.edu.pk

**Keywords:** *Oenanthe javanica*, blanching, secondary metabolites, RNA-seq analysis, vitamins, antioxidant activity

## Abstract

Field blanching is a process used in agriculture to obtain sweet, delicious, and tender stems of water dropwort by obstructing sunlight. The nutritional and transcriptomic profiling of blanched water dropwort has been investigated in our previous studies. However, the effect of blanching on the production of secondary metabolites and different vitamins in water dropwort has not been investigated at the transcriptomic level. This study explored the transcriptomic variations in the phenylpropanoid biosynthesis, flavonoid biosynthesis, and different vitamin biosynthesis pathways under different blanching periods in the water dropwort stems (pre-blanching, mid-blanching, post-blanching, and control). The results show that polyphenol and flavonoid contents decreased; however, the contents of vitamins (A, B1, B2, and C) and antioxidant activity increased significantly after blanching. Furthermore, the Kyoto Encyclopedia of Genes and Genomes (KEGG) enrichment analysis of blanched water dropwort showed the downregulation of many important genes involved in phenylpropanoid and flavonoid biosynthesis pathways, and the downregulation of these genes might be the reason for the reduction in polyphenol and flavonoid contents. We also examined and highlighted the genes involved in the higher vitamin content, antioxidant activity, pale color, tenderness, and sweetness of the blanched stem of water dropwort. In conclusion, the present study explored the role of phenylpropanoid and vitamin biosynthesis, and it will provide a basis for future investigation and application in the blanch cultivation of water dropwort.

## 1. Introduction

Vegetables are important sources of different vitamins, minerals, dietary fibers, carbohydrates, and natural antioxidants, such as phenolics and flavonoids [1,2,3]. *Oenanthe javanica* (also known as water dropwort) is an important aquatic vegetable and belongs to the *Apiaceae* family. It is commonly cultivated in different countries, such as Japan, Korea, China, and Thailand [4,5,6]. It is an excellent source of different minerals, vitamins, dietary fiber, and secondary metabolites. It is used as traditional Chinese medicine for the treatment of many diseases [6,7,8]. Phytochemicals extracted from water dropwort have several pharmacological activities: antioxidant activities, anti-inflammatory, anti-arrhythmic, anti-hepatitis B virus, hepatoprotective, antidiabetic, and anticancer [5,7,9]. Due to its particular aroma and flavor, it is used for garnishing different dishes and salads. The Chinese use the blanched stem of water dropwort with rice and other dishes in a fried or boiled form [2,7,10,11].

Blanching is a process used in agriculture to obtain sweet, delicious, and tender stems of water dropwort by obstructing sunlight. In the agriculture field, this technique is applied for the blockage of sunlight, which inhibits the production of chlorophyll. As a result of blanching, the water dropwort stem turns pale white, sweet, and tender [11,12,13]. Different blanching techniques are used to cover the water dropwort in the soil, and the deep planting method is identified as the most appropriate technique for the blanching of water dropwort [2,13,14,15]. Our previous study reported that blanching had enhanced the nutritional value in the form of minerals, vitamins, and antioxidant capacities of the different water dropwort cultivars. Moreover, V11E0012 (Jianglingye shuiqin) was identified as the most suitable cultivar for blanching [2]. We also reported that the higher antioxidant capacity of blanched water dropwort was due to the higher content of vitamins and soluble sugars [2]. Another study by Kumar et al. (2021) reported that a significant number of the genes were found downregulated in photosynthesis-related pathways that eventually reduced the chlorophyll content in water dropwort due to blanching [10]. Our previous study also reported that blanched samples contained the upregulation of many genes in the plant hormone signal transduction pathways and transcription factors, such as ERF, bHLH, MYB, and zinc finger. We also highlighted the role of these pathways and genes in the stimulating length, development, and stress tolerance in the blanched stem of water dropwort [10]. However, the influence of blanching on the production of secondary metabolites and vitamins in the water dropwort has not been investigated at the transcriptomic level.

Phenylpropanoids are a major category of secondary metabolites, including flavonoids, isoflavonoids, catechin, stilbenes, coumarins, lignin, and lignocellulose [16,17]. These are present in different vegetables, fruits, herbs, and nuts and possess antioxidant, anti-inflammatory, anticancer, antiviral, and antitumor activities. These also provide defense against cardiovascular diseases and strengthen the immune system [17,18]. Phenylpropanoids improve the tolerance of plants against different environmental stresses. These secondary metabolites provide particular taste, color, and texture to the food in the consumable form. Lignin is the category of phenylpropanoids, which have a role in the structural integrity of plant cell walls and stem strength [18,19,20].

The current study explored the transcriptomic variations in the phenylpropanoid biosynthesis, flavonoid biosynthesis, and different vitamins biosynthesis pathways. We then investigated the genes involved in the pale white color, tenderness, sweetness, and higher antioxidant activity of the blanched stem of water dropwort. This research provides understandings of phenylpropanoid and vitamins biosynthesis in water dropwort under blanched conditions.

## 2. Materials and Methods

### 2.1. Experimental Conditions and Blanching with the Deep Planting Method

In this experiment, the V11E0012 (Jianglingye Shuiqin) cultivar of water dropwort (the origin of this cultivar is Jiangling county in Hubei province, China) was used for the physiological and transcriptomic analyses. A field with a fertile soil and proper irrigation was used. The matured water dropwort stems were cut into 3.3–3.5 cm long stem segments (each segment had to have a stem node). For the germination of new plants, the cut stems were kept in a cold ventilated area and water was sprinkled daily. Sunshade nets were used to cover the plants for the retention of moisture; after 7–8 days, new shoots were germinated. Newly germinated shoots were moved to seedbeds, concealed with a 1–1.5 cm layer of the soil, and covered with the sunshade net. A month later, the plants around 10 cm in height were used for the plantation with the hill planting method. The distance between the hills was around 10 × 10 cm, and every hill had four plants. When the heights of the plants were ∼30 cm, blanching was applied [2,15,21].

When the water dropwort height reached up to 30 cm, the plants were removed from each hill and bound in bunches. Each bunch, containing 30 plants, was dumped in soil 20 cm deep soil, then collected after 40 days. The experiment was based on a factorial RCBD model [2,21]. Stem was used for analysis, all the data were analyzed in triplicates, and there were four time-points for stem sampling: pre-blanching, mid-blanching (after 20 days), post-blanching (40 days), and control (grown under normal conditions in the field for 40 days).

### 2.2. Assays for Total Antioxidant Capacity

For antioxidant capacity determination, 0.2 g of fresh stem samples were homogenized in 1.8 mL of 0.1 M PBS and centrifuged at 3500× *g* for 10 min. The supernatant was used for the determination of total antioxidant capacity. A commercially available kit of Nanjing Jiancheng Bioengineering Institute, China (total antioxidative capability assay kit, A015-1) was used, and its absorbance level was determined at the wavelength of 520 nm [22,23].

### 2.3. Determination of Polyphenol and Flavonoid Contents and DPPH Scavenging Activity

#### 2.3.1. Extraction Protocol

About 0.25 g of freeze-dried stem samples were homogenized in 16 mL of 10 mM ammonium acetate/methanol (50:50, *v*/*v*). Then, the homogenate was placed for 15 min in the ultrasonic bath and centrifuged for 15 min at 10,000× *g*. The collected supernatant was filtered and then used to quantify the polyphenols, flavonoids, and DPPH [2,24].

#### 2.3.2. Polyphenol Quantification

Gallic acid (GAE) is used as a standard for polyphenol quantification. The Folin-Ciocalteu method given by Sarker and Oba, (2020) was used [25]. A total of 2.5 mL of Folin-Ciocalteu reagent (1:10) was mixed with 500 µL of plant extract, then 2 mL of sodium carbonate decahydrate (0.75 g/mL) solution was added, and incubated for 15 min at 45 °C. Then, the mixture was retained at room temperature for 60 min; after that, the absorbance was calculated at 760 nm, and the polyphenol contents were presented as GAE/100 g.

#### 2.3.3. Flavonoid Quantification

Catechin (CE) is used as a standard for flavonoid quantification. The aluminum chloride method described by Sarker and Oba, (2019a) and Kumar et al. (2020) was used [4,26]. About 150 µL of sodium nitrite (500 mg/mL) solution was mixed with 2 mL of ddH_2_O and 0.5 mL of plant extract. The mixture was retained at room temperature for 5 min; after that, 150 µL of aluminum chloride (1 g/mL) solution, 1 mL sodium hydroxide (1 M), and 1.2 mL ddH_2_O were added. Finally, the absorbance was calculated at 510 nm and was presented as CE/100 g.

#### 2.3.4. DPPH Scavenging Activity Analysis

DPPH (2,2-diphenyl-1-picryl-hydrazyl-hydrate) scavenging activity was measured with the protocol described by Sarker and Oba (2020) and Kumar et al. (2021b) [2,25]. Around 1 mL of plant extract was mixed with 2.5 mL of 0.1 mM DPPH solution. The tube was vortexed and incubated for a half-hour at 25 °C. AfSter that, the DPPH decolorization was measured at the wavelength of 517 nm. Initially, a dark purple blank DPPH solution without extract was measured. The results of DPPH were presented against GAE, and the percentage of inhibition was described with the following correlation:

(1) $$\%\;\mathrm{Inhibition}=\frac{\left(A\;\mathrm{blank}-A\;\mathrm{sample}\right)}{A\;\mathrm{blank}}\times 100$$

### 2.4. Determination of Vitamins

#### 2.4.1. Vitamin A, B1, and B2 Quantification

For the quantification of vitamins (A, B1, and B2), around 1 g of fresh stem samples were crushed and mixed with 9 mL of 0.01 M PBS. The homogenates were sonicated for about 60 s and centrifuged for about 10–12 min at 5000× *g*. The supernatant was collected and used for the analysis of vitamins A, B1, and B2 using commercial ELISA kits manufactured by Shanghai Jingkang Bioengineering Co., Ltd. © 2022 (Shanghai, China) (gelatins.com.cn, accessed on 12 January 2021) [2]. The protocols for vitamin A, B1, and B2 were the same. Each kit had its own 96 wells, pre-coated microwell plates, standards, and chemicals. Each sample (50 µL) was added to the microplate wells pre-coated with antibodies. After the addition of the samples, the microplates were gently mixed and incubated for 45 min at 37 °C. The liquid was discarded, and each well was washed four times with 350 µL of washing solution and dried on absorbent paper. Approximately 50 µL of biotinylated anti-IgG was added and incubated for 30 min at 37 °C, then the washing step was again performed. After washing, 50 µL of streptavidin-HRP was added to each well, which was incubated at 37 °C for 15 min. The washing step was repeated, and 50 µL of chromogen solution A and chromogen solution B were added stepwise. The mixtures were incubated at 37 °C for 15 min. After incubation, 50 µL of termination solution was added to each well. Finally, OD was determined at 450 nm [2].

#### 2.4.2. Vitamin C Quantification

For analysis of vitamin C, 0.05 g of fresh samples of the stem were homogenized with 450 μL 0.1 M of PBS and centrifuged for 10 min at 4500× *g*. The supernatant was processed further to determine vitamin C content using the kit of Nanjing Jiancheng Bioengineering Institute, Nanjing, China (A009). Approximately 150 µL of the supernatant was mixed with 450 µL of reagent I (available in the kit), left to stand for 15 min at room temperature, and centrifuged at 4000× *g* for 10 min. The supernatant was then collected and processed further with the reagents of the kit, and absorbance was measured at 536 nm [2].

### 2.5. Statistical Analysis

For the statistical analysis, SPSS 25.0 statistical program (IBM Crop., Armonk, NY, USA) was used. A significant difference (*p* ≤ 0.05) was determined using Tukey tests, and the difference was presented by different letters in the figures and tables. For the figures, GraphPad Prism 7 (San Diego, CA, USA) was used. All the experiments were conducted in triplicates, and the data are presented as mean ± standard error (S.E).

### 2.6. Transcriptomic Analysis

#### 2.6.1. RNA Extraction, Library Construction, and Illumina Sequencing

Total RNA was extracted from the fresh stem of control samples and pre-, mid-, and post-blanched samples with Trizol (Invitrogen, Santa Clara, CA, USA). The RNA quality and quantity were determined with BioDrop uLite (80-3006-51). The cDNA library construction and transcriptomic sequencing were performed by *Biomarker* Technologies (Beijing, China) (http://www.biomarker.com.cn/, accessed on 4 February 2021) on the Illumina HiSeq 2000 platform [4,10,27].

#### 2.6.2. Quality Control and Transcriptomic Assembling

Raw data in the FASTQ format were processed through in-house Perl scripts. The clean data were achieved by eliminating reads having ploy-N, adapters, and low-quality reads from the raw data. Simultaneously, Q30 percentage, GC, and duplication level of a sequence of the clean data were measured. All the analyses downstream were based on high-quality clean data. The left files (read1 files) from all samples/libraries were assembled into a single big left.fq file and all files from the right (read2 files) were pooled into a single big right.fq file. The transcriptomic assembly was based on the left.fq and right.fq by using Trinity with min-kmer-cov fixed to 2 by default, and all other parameters were also set by default [4,10,27]. We submitted the transcriptomic data to the Sequence Read Archive (SRA) database of NCBI, and the SRA accession number is PRJNA722062.

#### 2.6.3. DEGs Identification and Their Functional Annotations

The gene expression levels of every sample were calculated using RSEM [28]. The expression analysis of genes for four-time points was accomplished by using the DESeq R package (1.10.1). For determination of significant differential expression, the threshold was fixed at a *p*-value smaller than 0.05 and a Log2 (fold change) greater than 1 [4,10,27,29]. Gene ontology (GO) enrichment analysis was performed using topGO R packages, and pathway enrichment analysis was performed by the KOBAS software [30,31].

### 2.7. Quantitative Real-Time PCR Analysis

Total RNA was extracted from the fresh stem of control samples and pre-, mid-, and post-blanched samples with Trizol (Invitrogen, Santa Clara, CA, USA). The SuperScript III reverse transcriptase (Invitrogen, Grand Island, NY, USA) kit was used for reverse transcription reactions. qRT-PCR analysis was performed by the CFX96 real-time PCR system and Bio-rad with TB Green Premix Ex Taq™ II (Tli RNaseH Plus) (TaKaRa Bio. Co., Beijing, China). Primer3 software was used in the design of primers. *Actin* (*c41123.graph_c0*) was used as the internal control. The list of primers for the *Actin* (internal control) and ten selected genes are given in Table 1. The protocol of Kumar et al. (2020 and 2021a) was used for amplification [4,10]. All the qRT-PCR analyses were performed in biological triplicates. Relative expression levels of the genes were analyzed by the 2^−ΔΔCt^ method [32,33].

## 3. Results

### 3.1. Polyphenol and Flavonoid Contents and DPPH Analysis

During blanching, different trends were observed in polyphenol and flavonoid analysis. Total contents of polyphenols and flavonoids increased significantly in the mid-blanched period; however, a significant decrease was observed in the post-blanched samples compared to the pre-blanching samples. The control samples showed reduced polyphenol and flavonoid contents compared with the pre-blanching samples (Table 2).

DPPH (2,2-diphenyl-1-picryl-hydrazyl-hydrate) scavenging activity was decreased in the mid-blanched samples. Conversely, a slight increase was observed in the post-blanched samples. Post-blanched samples exhibited a 10% increment in DPPH activity (Table 2). Likewise, the total antioxidant capacity of the blanched stem increased significantly with the increase in the blanching period (*p* < 0.05), and the post-blanching samples showed the highest increments compared with their counterparts However, the control samples showed an insignificant decrease in the total antioxidant capacities in comparison with the pre-blanched samples (Table 2).

### 3.2. Vitamin Analysis

The contents of vitamins A, B1, B2, and C were increased significantly with the blanching period, and the highest increments were observed in the post-blanched stem samples (*p* < 0.05). Post-blanched samples showed a 44.7% increment in vitamin A compared to the pre-blanching samples. The contents of vitamins B1 and B2 increased by 49.6% and 33.8%, respectively, in the post-blanched stems than in the pre-blanched stems. However, the contents of vitamin B1 and B2 increased insignificantly in the control samples compared to the pre-blanching samples. Likewise, vitamin C content was increased by 76.5% in the post-blanched samples compared to the pre-blanched samples. By contrast, the control samples showed lower vitamin C contents than their pre-blanching samples (Table 3).

### 3.3. Transcriptomic Sequencing and Assembly

A comparative transcriptomic analysis showed that the Q30 score was above 93.5%, the mapped proportion was above 78.6%, the GC content was above 44.2%, the generated transcripts were 224,926, and unigenes were 62,336 (Table S1) [10].

### 3.4. Differentially Expressed Gene (DEG) Analysis of Blanched Water Dropwort

**Figure 1:**
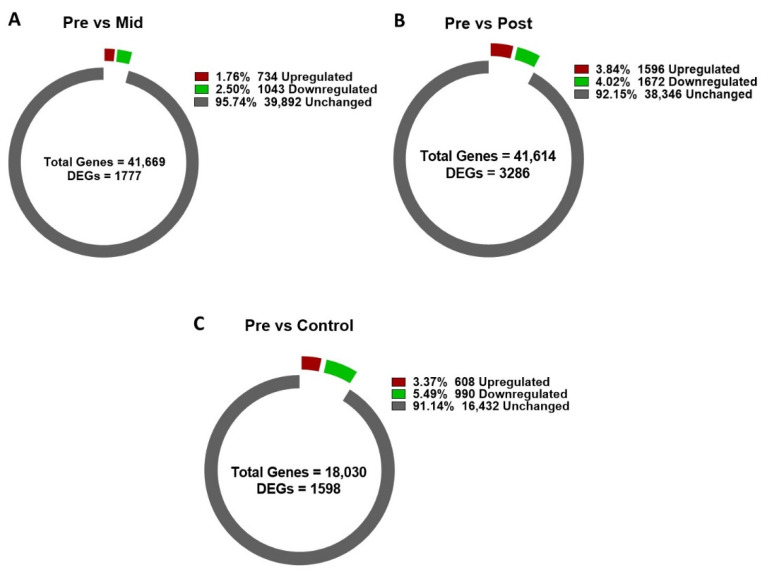
Differentially expressed genes (DEGs) in water dropwort stem under blanched and control conditions. (**A**) Mid−blanched, (**B**) post−blanched, and (**C**) control conditions. Pre−blanching samples were used as a reference for comparison.

The reference for the comparison was pre-blanched samples. Mid-blanched samples showed 1777 DEGs, comprising 734 upregulated DEGs. Post-blanched samples have 3268 DEGs, of which 1596 were found upregulated [10]. Whereas 1598 DEGs were found in the control samples, which contained 608 upregulated (Table S2; Figure 1). DEGs in the heat map were distributed into three groups on the basis of expression level (Figure S1); we found that Group A was more downregulated in the post-blanched samples. DEGs of Group B were mostly found upregulated in post-blanched samples; however, a high level of downregulation was depicted in the mid-blanched period, followed by pre-blanching samples. Moreover, Group C showed higher upregulation in mid and post-blanched samples than in pre-blanching and control samples (Figure S1).

DEGs were BLAST against public databases, and from that, we found that mid-blanching had 1662 annotated DEGs, post-blanching had 2985, and the control sample had 1493 annotated DEGs (Figure S2).

### 3.5. Kyoto Encyclopedia of Genes and Genomes (KEGG) Pathway Enrichment Analysis

KEGG IDs were allotted to 326 DEGs in mid-blanched samples, 614 DEGs in post-blanched samples, and 241 DEGs in control samples. These DEGs were further classified into 96, 113, and 91 pathways, respectively. The phenylpropanoid biosynthesis, flavonoids biosynthesis, ascorbate and aldarate metabolism, thiamine metabolism, riboflavin metabolism, vitamin B6 metabolism, and nicotinate and nicotinamide metabolism pathways showed the highest number of DEGs in the post-blanched samples (Table S3).

#### 3.5.1. Phenylpropanoid Biosynthesis Pathway

**Figure 2:**
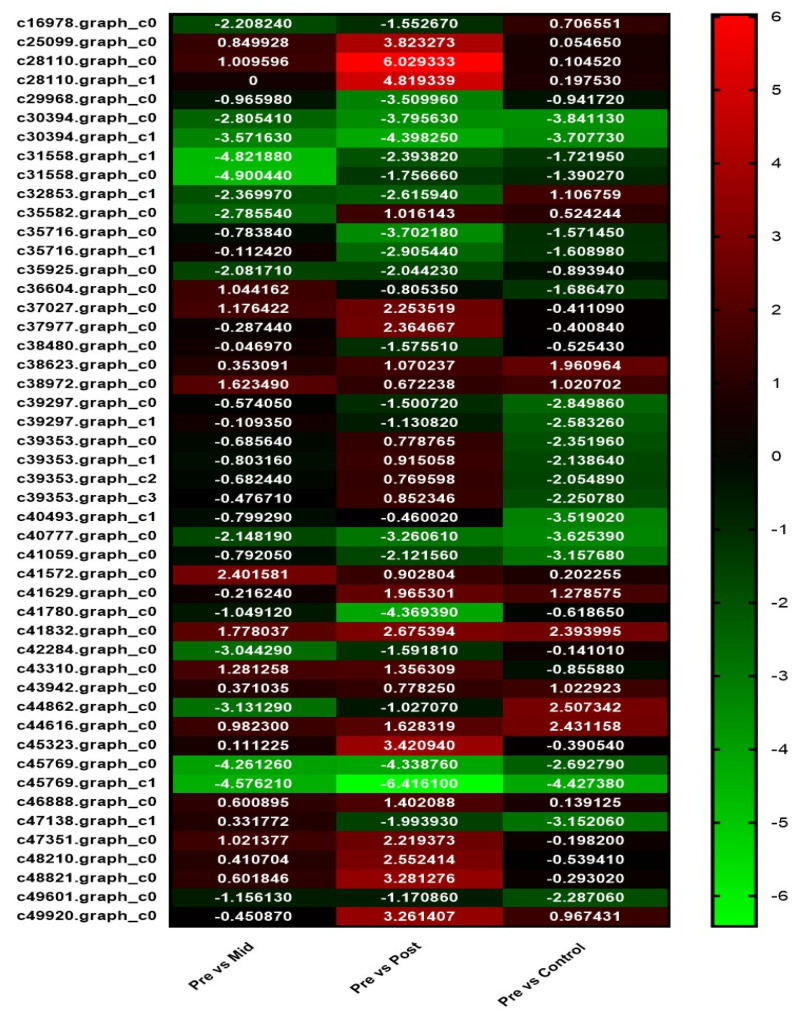
Heat map of DEGs involved in phenylpropanoid biosynthesis pathway in the water dropwort under mid−blanched, post−blanched, and control conditions.

The results of the phenylpropanoid biosynthesis pathway showed that the number of dysregulated genes in post-blanched stem samples were higher than in the other counterparts (Figure 2; Table S4). In total, 25 dysregulated DEGs were identified in the post-blanched, 16 in the mid-blanched, and 18 in the control samples. Among these dysregulated genes, 14 were found upregulated in the post-blanching samples, whereas 3 in the mid-blanching and 3 in the control samples were found to be upregulated. By contrast, the number of downregulated DEGs were 11, 13, and 15 in the post-blanched stem, mid-blanched stem, and control samples of the stem, respectively.

#### 3.5.2. Flavonoid Biosynthetic Pathway

**Figure 3:**
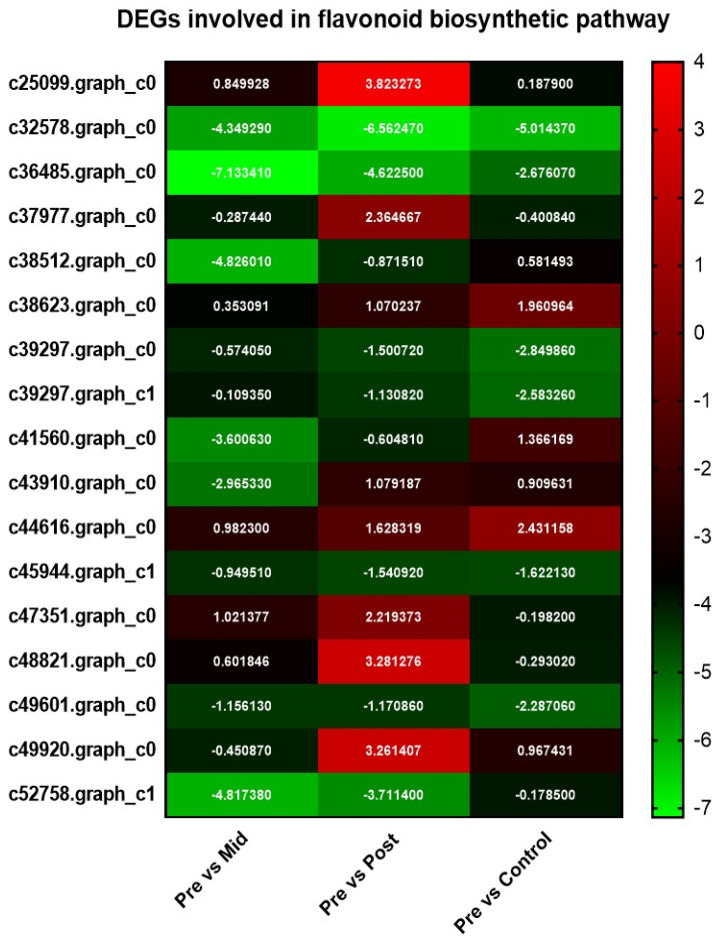
Heat map of DEGs involved in flavonoid biosynthesis pathway in the stem of water dropwort under mid−blanched, post−blanched, and control conditions.

Post-blanched samples showed eight DEGs, in which five were found upregulated, and three were found downregulated. Mid-blanched samples, by contrast, showed six dysregulated genes, and all were downregulated. The control samples showed eight DEGs, in which six were downregulated and two were upregulated (Figure 3; Table S4).

#### 3.5.3. Upregulated DEGs in the Biosynthesis of Vitamins

**Figure 4:**
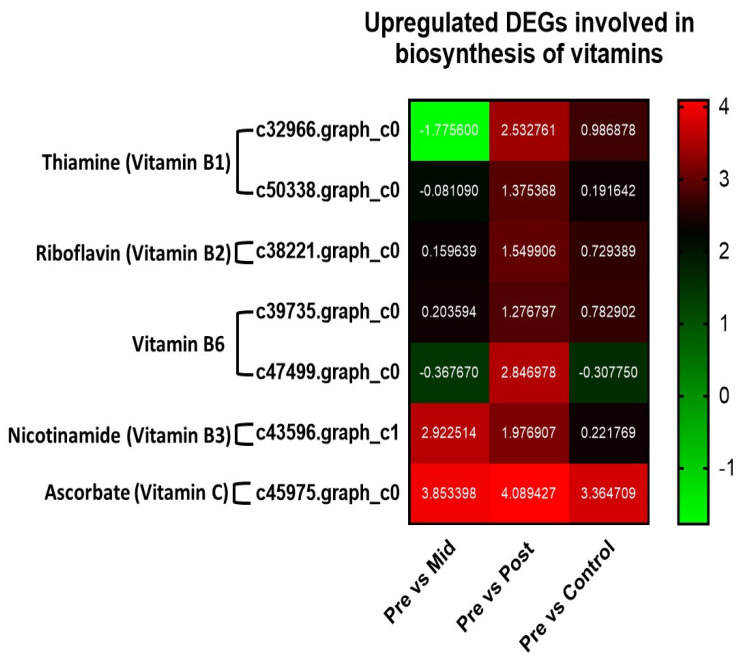
Heat map of upregulated DEGs involved in vitamin biosynthetic pathways in water dropwort under mid−blanched, post−blanched, and control conditions.

In vitamin biosynthesis, we found upregulation of genes in post-blanched samples compared to the other counterparts (Figure 4; Table S4). In the thiamine (vitamin B1) biosynthesis pathway, we found two upregulated genes (*c32966.graph_c0* and *c50338.graph_c0*) in the post-blanched samples compared to the other counterparts. Similarly, the riboflavin (vitamin B2) biosynthesis pathway showed only one overexpressed gene (*c38221.graph_c0*) in the post-blanched samples.

We found two upregulated genes *(c39735.graph_c0* and *c47499.graph_c0*) in the vitamin B6 biosynthesis pathway. Similarly, one gene (*c43596.graph_c1*) was found overexpressed in the nicotinamide (vitamin B3) biosynthetic pathway, and one gene (*c45975.graph_c0*) in the ascorbate (vitamin C) biosynthetic pathway in the post-blanched samples in comparison to the other counterparts (Figure 4; Table S4).

### 3.6. Validation of DEGs

**Figure 5:**
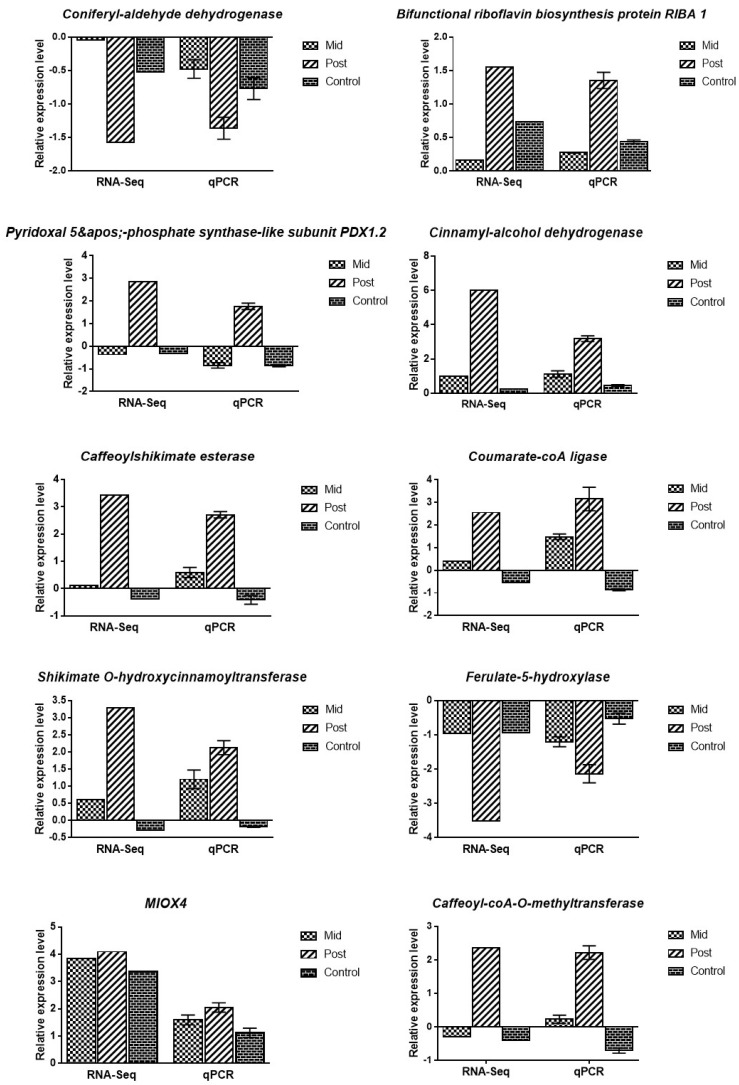
qRT−PCR analysis of ten representative genes for the confirmation of RNA−seq data. The bars on the qPCR data depict the means ± SD.

QRT-PCR analysis was conducted on 10 selected genes from different pathways tovalidate the RNA-seq data; control, pre-, mid-, and post-blanching were used in the qRT-PCR analysis. The expression profiles of these genes under qRT-PCR showed the same trends as in the RNA-seq analysis, which validated its reproducibility and reliability (Figure 5).

## 4. Discussion

A previous study by Kumar et al. (2021b) described that blanching had significantly promoted stem length as well as fresh and dry biomass. By contrast, a significant decrease was observed in the root length, branches, and leaves during the blanching period [2]. Previous studies reported that ethylene accumulation during submergence, flooding, and deep planting encourages stem elongation and leaf senescence and slows down photosynthetic carbon fixation [34,35]. Another study conducted by Kumar et al. (2021a) also showed the overexpression of various ethylene-associated genes under blanched conditions [10].

Total phenolics and flavonoid improve the tolerance of plants against different environmental stresses [18]. However, a study conducted by Wang et al. (2020) reported reduction of total phenolics and flavonoid contents under shade treatments [36], indicating the importance of high light intensity in polyphenol and flavonoid biosynthesis. In the current study, we found that the blanching caused a significant reduction in the polyphenol and flavonoid contents, and the post-blanching samples showed the lowest contents of polyphenol and flavonoids. Kumari et al. (2009) revealed that the downregulation of genes involved in the phenolic acid synthesis pathway under low light intensity is the reason for reducing total phenols [37]. In the current study, the control samples also showed insignificant reduction in total phenolic and flavonoid contents. Deng et al. (2020) reported that under natural conditions, the total phenolic and flavonoid contents declined with the maturation and growth of the plant, corresponding to declined antioxidant activities [38].

In the present study, more reduction in polyphenol and flavonoid contents were detected in blanched samples compared to the control. However, higher numbers of downregulated genes were found in the control samples compared to the blanched samples. Deng et al. (2020) reported that polyphenols/flavonoid genes were mainly enriched in the different DEG groups of sweet potato, the expression levels of some activators or transcription factors (TFs) (*IbMYB1*, *IbMYB3*, and *IbbHLH2*) and structural genes (*IbF3H*, *IbF3’H*, *IbANS*, and *IbGGT1*) were significantly decreased between different growth stages, and caused the reduction in the expression of polyphenol/flavonoid genes [38]. In the current study, a higher number of downregulated DEGs in polyphenol/flavonoid genes in the control samples of the stem might be due to the downregulation of MYB and bHLH TFs. In our previous study, we found 15 upregulated genes related to MYB and 17 upregulated genes related to bHLH in blanched samples. By contrast, the control samples had only five upregulated and seven downregulated genes in MYB and two upregulated and nine downregulated genes in bHLH [10]. Therefore, more downregulation of phenylpropanoid and flavonoid pathway genes might be due the downregulation of these TFs. Moreover, based on the assay of total phenols and flavonoids, we suggest that post-translational modifications, protein–protein interactions, and phenotype may have influenced the total contents of phenols and flavonoids [4,39].

Different studies have reported that biotic and abiotic stresses increase phenylpropanoid biosynthesis [40,41]. We found upregulation of genes, such as *coumarate-coA ligase*, *shikimate hydroxycinnamoyl transferase*, *caffeic acid 3-O-methyltransferase*, *cinnamyl-alcohol dehydrogenase*, and *caffeoylshikimate esterase* in the post-blanched samples. By contrast, these same genes were found downregulated in the control conditions. Previously, *4-coumarate-coA ligase* showed a positive response against UV light in he wheat [42] and chilling in peach [43]. Similarly, *hydroxycinnamoyl transferase* also plays a significant role in plant protection against abiotic stress [44]. Furthermore, *caffeoylshikimate esterase* exhibited a role in phospholipid repair under oxidative stress [45]. *Caffeoyl-coA-O-methyltransferase* has been found to be upregulated under salt stress in foxtail millet, tomato root, and the leaves of *Puccinellia tenuiflora* [46,47,48]. All these reports favor our findings, and we speculate that phenylpropanoid and flavonoid biosynthesis has positive role in the stress response in the blanch cultivation of water dropwort.

Phenolic compounds can play a vital role in flavonoid production and a significant role in lignin biosynthesis [49,50]. The role of *anthocyanidin synthase* in the anthocyanin-biosynthesis pathway has been characterized in many plants, such as Arabidopsis, apple, and strawberry [51,52,53]. The silencing of this gene in the apple caused almost complete blockage of anthocyanin biosynthesis [53]. Another report by Ben-Simhon et al. (2015) mentioned that anthocyanin-less fruit had a high susceptibility to sunburns and browning [54]. Similarly, another report by Rafique et al. (2016) mentioned that *anthocyanidin synthase* caused the biosynthesis of anthocyanin, which developed a bitter taste [55]. We found downregulation of *anthocyanidin synthase* and *flavanone 3-hydroxylase* genes in post-blanching samples. These genes may play an important role in the white color and better taste of the water dropwort stem. We also found that genes involved in lignin biosynthesis, such as *ferulate-5-hydroxylase* and *coniferyl-aldehyde dehydrogenase* were downregulated only in the blanched samples. Sibout et al. (2002) reported that *ferulate-5-hydroxylase* has a role in S-lignin deposition and its overexpression enhanced the S-lignin level in *Arabidopsis thaliana* [56]. *Coniferyl aldehyde dehydrogenase*, was involved in the biosynthesis of ferulic acid in *Zea mays*, an essential structural component in the cell wall and increase its strength and rigidity [57]. Two other genes, *cinnamoyl-coA reductase* and *peroxidase* were also found to be downregulated in both the blanched and the control samples. *Cinnamoyl-coA reductase* and *peroxidase*, are involved in lignin polymerization, and suppression of these genes caused a reduction in lignin level by 50% [58]. Jia et al. (2015) described that the taste of celery is affected by cellulose and lignin contents [19]. The downregulation of the genes mentioned, particularly in blanched water dropwort, might be the reason for the tender stem of water dropwort after blanching. Low levels of flavonoids and phenolics provide a better taste and low bitterness [59]. Therefore, we assume that the sweet or delicious taste of blanched water dropwort is due to reduced flavonoids and phenolics. We also presume that the better taste of blanched water dropwort might be due to the downregulation of genes involved in lignin biosynthesis. Overall, we can speculate that many upregulated genes in the phenylpropanoid and flavonoid biosynthesis pathways may help to stand in the blanching conditions. Furthermore, the pale white color, tenderness, and better taste of blanched water dropwort may be due to the downregulation of genes involved in the phenylpropanoid and flavonoid biosynthesis pathways.

Generally, polyphenols in plants are associated with redox properties and antioxidant activities. The production of polyphenols increased under abiotic stress conditions through the regulation of the overproduction of reactive oxygen species (ROS) [60,61,62]. In the current study, DPPH scavenging activity in the post-blanched samples slightly increased compared with the pre-blanched samples. Likewise, a significant increment in the total antioxidant capacities of blanched water dropwort was observed, and the post-blanched samples exhibited the maximum antioxidant capacities. Kumar et al. (2021b) reported an increase in total antioxidant capacity among five cultivars of blanched water dropwort [2]. Samuolienė et al. (2011) reported an increase in DPPH activity in the dark and mentioned that this increase was due to the higher level of polyphenols, ascorbic acid, and alpha-tocopherol [63].

Although the polyphenol and flavonoid contents decreased in blanched water dropwort, total antioxidant capacities still increased due to the non-enzymatic antioxidant system involving β-carotene, vitamin B1, vitamin B2, vitamin C, α-tocopherol, and soluble sugars. These non-enzymatic compounds have antioxidant capabilities and assist in reducing ROS [62,64,65,66,67]. Our current study found that blanched water dropwort has higher antioxidant activities, which could positively help water dropwort and human health. In physiological studies, we found higher contents of vitamins (A, B1, B2, and C) in the post-blanched samples. Transcriptomic analysis in the present study also showed the upregulation of many genes involved in the biosynthesis of vitamins B1, B2, B3, B6, and C.

Thiamine (vitamin B1) was found to be involved in the response to several abiotic and biotic stresses, and it also acts as a cofactor for different enzymatic reactions [68]. Enhancing the thiamine content is among the important breeding objectives to overcome its deficiency in staple crops with low thiamine levels. We found the upregulation of genes (*thiamine thiazole synthase* and *cysteine desulfurases*) in the post-blanched samples. Previous studies revealed that *thiamine thiazole synthase* has a significant role in the biosynthesis of thiamine [69]. Similarly, *cysteine desulfurases* were also found to be involved in the biosynthesis of thiamin [70]. These genes may be responsible for the high content of thiamine in the blanched stem of water dropwort.

Riboflavin (vitamin B2) is essential for all living organisms. Plants are a major source of riboflavin for animals. Several reports showed that riboflavin enhances the accumulation of antioxidant compounds in plants, and it can help several enzymes in regulating H_2_O_2_ scavenging [71,72]. Deng et al. (2014) found that riboflavin in the tobacco plant increased drought tolerance [64]. The bifunctional riboflavin biosynthesis protein RIBA1 is the first enzyme involved in riboflavin biosynthesis, and mutant bifunctional riboflavin biosynthesis protein RIBA1 (*AtRIBA1*) showed a low level of riboflavin in Arabidopsis [73]. We also found upregulation of the *RIBA1* gene in the post-blanched samples of water dropwort, which might be the reason for the higher level of vitamin B2 in the blanched samples.

Vitamin B6 (pyridoxal phosphate) is an important cofactor for many enzymatic reactions and it also shows a response to oxidative stress in plants [74]. The biosynthetic pathway of vitamin B6 showed the upregulation of *Inorganic pyrophosphatase 1* and *Pyridoxal 5&apos;-phosphate synthase-like subunit PDX1.2* (*PDX12*) genes in the post-blanched samples. On the other hand, only *PDX12* was also found to be upregulated in the control samples. *Inorganic pyrophosphatase 1* was involved in the phosphate starvation response [75]. Therefore, we assume that *Inorganic pyrophosphatase 1* might also respond positively under blanching. 

Nicotinamide can enhance plant protection against different abiotic stresses [76]. One of the genes involved in the nicotinamide pathway, known as survival protein *surE*, plays a significant physiological role in the response to stress conditions [77]. In addition, it also showed a potential role in phosphate scavenging under stress conditions [78]. We found upregulation of the *surE* gene in post-blanching conditions, which might be involved in stress tolerance.

We found higher ascorbic acid content in our physiological studies under blanching. The vitamin C content was increased under etiolated conditions and played a significant role as an antioxidant to overcome oxidative stress in wheat [79]. Likewise, an increase was observed in the contents of ascorbic acid and α-tocopherol in the dark [63]. The content of vitamin C in fruits and vegetables can be influenced by various factors, such as genotypic differences, preharvest climatic conditions, cultural practices, maturity and harvesting methods, and postharvest handling procedures [80]. As we applied blanching, changes in the content of vitamin C was observed.

The *MIOX4* caused an increment in the content of ascorbic acid in *Arabidopsis* compared with the controls [81,82]. We also found upregulation of *MIOX4* in the blanched and control samples of water dropwort. Ascorbate oxidase has been proposed to be the major enzyme responsible for the enzymatic degradation of ascorbic acid. Ascorbate oxidase is a copper-containing enzyme that oxidizes ascorbic acid to dehydroascorbic acid in the presence of molecular oxygen [83]. Ascorbate oxidase is associated with rapidly growing regions in the plant and occurs bound to cell walls as well as a soluble protein in the cytosol [84]. Therefore, it might be possible that enzymes cause the enzymatic degradation of ascorbic acid, which ultimately causes the reduction in vitamin C in the control samples of water dropwort. Other reasons may be that post-translational modifications, protein–protein interactions, and phenotype influenced the vitamin C content. Therefore, we cannot dismiss the possibility. Further genetic–level investigations may be needed for confirmation. Etiolation can increase β-carotene content, a precursor of different vitamins and antioxidants [85]. Overall, we found upregulation of many genes involved in the biosynthesis of different vitamins, which might play an important role in the stress response and blanching. 

The present study showed that higher vitamins contents under blanched conditions increase the nutritional worth of water dropwort and may also assist in its growth, development, and stress tolerance. These vitamins could help in reducing the risk of different diseases and improve health [86,87]. Hence, we presume that higher level of vitamins could contribute to disease prevention and alleviation.

## 5. Conclusions

In conclusion, we found that polyphenol and flavonoid contents were decreased; however, the content of vitamins A, B1, B2, and C as well as total antioxidant activity increased significantly in the blanched stem of water dropwort. Furthermore, KEGG enrichment analysis of the blanched samples showed the downregulation of many important genes involved in phenylpropanoid and flavonoid biosynthesis pathways, and the downregulation of these genes might be the reason for the reduction in polyphenol and flavonoid contents. We also identified the upregulated genes in the vitamins biosynthesis pathways, which increased the vitamins content. We also identified genes involved in pale color, tenderness, and sweetness in the blanched stem of water dropwort. This study explored the role of phenylpropanoid and vitamins biosynthesis, though more studies are still required to validate its results. This research will provide a basis for research on the blanch cultivation of water dropwort.

## Figures and Tables

**Table 1:** List of primers for qRT-PCR analysis.

*Gene ID*	*Gene Name*	Tm
*c41123.graph_c0*	*Actin*	54.4
TTGAGCGGAGGGAGTACTAT		
GCGATGCCTTCTTCCAAATA		
*c38480.graph_c0*	*Coniferyl-aldehyde dehydrogenase*	55.0
AGTAGCAGAAGGTGACAAAG		
CAGCATAGTAACGCAAAGTG		
*c38221.graph_c0*	*Bifunctional riboflavin biosynthesis protein RIBA 1*	55.3
GGTTCTATGGCTCGATTACC		
TCATGCAACTGGAAAGGATT		
*c47499.graph_c0*	*Pyridoxal 5&apos;-phosphate synthase-like subunit PDX1.2*	54.9
TTGAAGCCCAGATTCTTGAA		
CATCACTTTCCTCACATTGC		
*c28110.graph_c0*	*Cinnamyl-alcohol dehydrogenase*	54.7
GTTGGAATAGGGTGCTTAGT		
CAGCACATAATAGAGGAGCA		
*c45323.graph_c0*	*Caffeoylshikimate esterase*	55.0
CACGTGGACTGGACTTATAG		
CTAGCTTTGCCCGAATATCT		
*c48210.graph_c0*	*Coumarate-coA ligase*	54.8
CCTACAATTTCTGATGCTGC		
GGATGCTATTCTTGCTCTCA		
*c48821.graph_c0*	*Shikimate O-hydroxycinnamoyltransferase*	53.4
TTATATTCACTGCCACGCCT		
CAAGGTTTGGGCACTGAAAA		
*c29968.graph_c0*	*Ferulate-5-hydroxylase*	54.4
ATGCTGATGATGGACCAGTT		
GCCCGGTCATATGAAAGGTA		
*c45975.graph_c0*	*Inositol oxygenase 4 (MIOX4)*	55.4
GCCAAAGAAAACGGGACTAC		
CAACTCGGACTTTGCTCTTG		
*c37977.graph_c0*	*Caffeoyl-coA-O-methyltransferase*	54.9
TTTGCCCGTTCTTGATCATA		
CTCGATCACAAAGTCTCTGT		

**Table 2:** Blanching effect on the contents of total polyphenols, flavonoids, DPPH, and total antioxidant capacity (T-AOC) in the stem of water dropwort.

Treatment	TPC mg GAE/ 100 mg FW	TFC mg CAE/ 100 mg FW	%DPPH Scavenging Activity	T-AOC (U/mg Protein)
Pre	447.1 ± 27.1 ^b^	220.2 ± 19.3 ^b^	51.1 ± 4.4 ^ab^	1.04 ± 0.11 ^a^
Mid	475.7 ± 8.9 ^c^	236.4 ± 20.7 ^b^	44.9 ± 2.4 ^a^	3.88 ± 0.24 ^b^
Post	307.9 ± 26.3 ^a^	126.3 ± 10.3 ^a^	56.3 ± 3.5 ^ab^	4.29 ± 0.39 ^b^
Control	386.0 ± 30.2 ^bc^	168.1 ± 13.1 ^b^	63.1 ± 4.7 ^b^	0.93 ± 0.06 ^a^

Different letters indicate a significant difference (*p* < 0.05) among the treatments according to the Tukey test. Values are means ± SE.

**Table 3:** Effect of blanching on the content of vitamins in the stem of water dropwort.

Treatment	Vitamin A µg/g FW	Vitamin B1 µg/g FW	Vitamin B2 µg/g FW	Vitamin C mg/g FW
Pre	0.680 ± 0.05 ^a^	1.065 ± 0.06 ^a^	0.870 ± 0.05 ^a^	0.068 ± 0.004 ^b^
Mid	0.755 ± 0.03 ^a^	1.390 ± 0.03 ^b^	1.054 ± 0.04 ^b^	0.106 ± 0.006 ^c^
Post	0.984 ± 0.04 ^b^	1.593 ± 0.06 ^c^	1.164 ± 0.09 ^b^	0.120 ± 0.005 ^d^
Control	0.657 ± 0.03 ^a^	1.111 ± 0.07 ^a^	0.885 ± 0.06 ^a^	0.024 ± 0.001 ^a^

Different letters indicate a significant difference (*p* < 0.05) among the treatments according to the Tukey test. Values are means ± SE.

## Data Availability

The datasets generated and analyzed during the current study are available in the National Center for Biotechnology Information (NCBI) repository, PRJNA722062 (https://www.ncbi.nlm.nih.gov/bioproject/PRJNA722062, accessed on 14 April 2021).

## Supplementary Materials

The following are available online at https://www.mdpi.com/article/10.3390/antiox11030470/s1, Figure S1: Cluster analysis of DEG profiles, data were divided into 3 data sets; Figure S2: Functional annotations of DEGs against public databases; Table S1: Summary of mapping transcriptome reads to the reference sequence and transcriptomic assembly; Table S2: Differentially expressed genes in control and blanched samples; Table S3: KEGG pathways; Table S4; Targeted pathways and their corresponding DEGs.

## Abbreviations

PBS	Phosphate-buffered saline
Pre-blanching	Before treatment
Mid-blanching	Blanched for 20 days
Post-blanching	Blanched for 40 days
Control plants	Grown under normal conditions in the field for 40 days
GAE	Gallic acid
CE	Catechin
TPC	Total polyphenol content
TFC	Total flavonoid content
T-AOC	Total antioxidant capacity
ROS	Reactive oxygen species
Q30 percentage	Quality score > 99.9%
RSEM	RNA- seq by expectation maximization
KOBAS	KEGG orthology-based annotation system
DPPH	2,2-diphenyl-1-picryl-hydrazyl-hydrate
RIBA1	Bifunctional riboflavin biosynthesis protein
*PDX12*	*Pyridoxal 5&apos*; -*phosphate synthase-like subunit PDX1.2*
TFs	Transcription factors
