# Supplementary material for: Influence of Blanching on the Gene Expression Profile of Phenylpropanoid, Flavonoid and Vitamin Biosynthesis, and Their Accumulation in Oenanthe javanica
Source: Antioxidants (Basel). 2022 Feb 26;11(3):470. doi: 10.3390/antiox11030470 (PMC8944621; doi:10.3390/antiox11030470)
Supplement: Supplementary file 1 [file antioxidants-11-00470-s001.zip › Supplementary figures.pdf]

Supplementary Figures

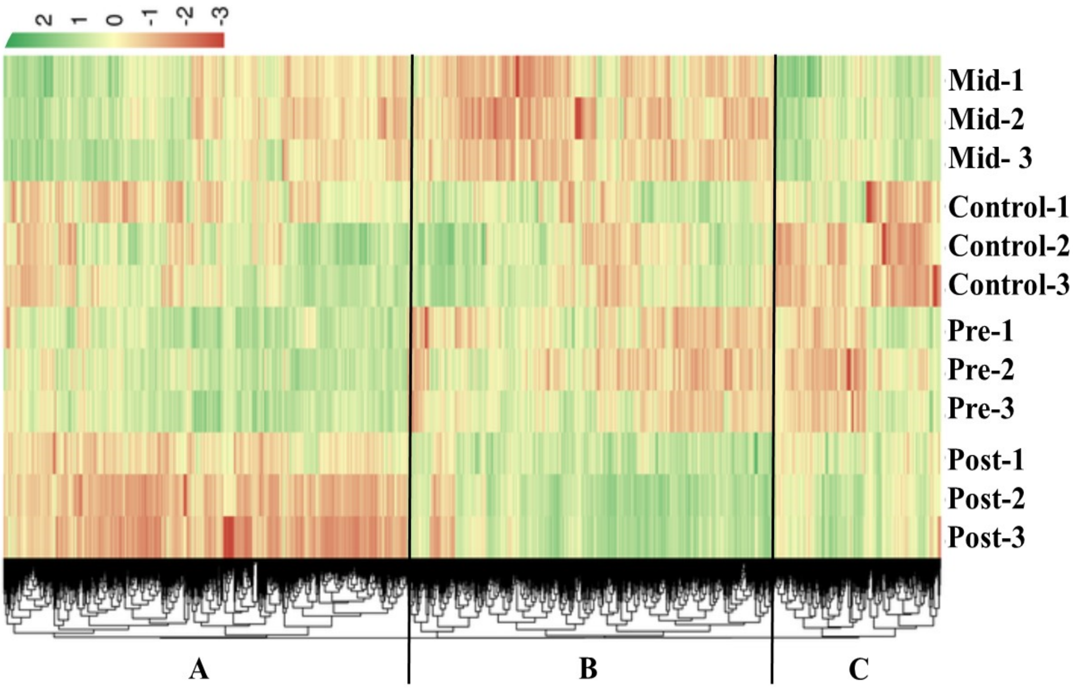

Figure S1. Cluster analysis of DEG profiles, data were divided into 3 data sets

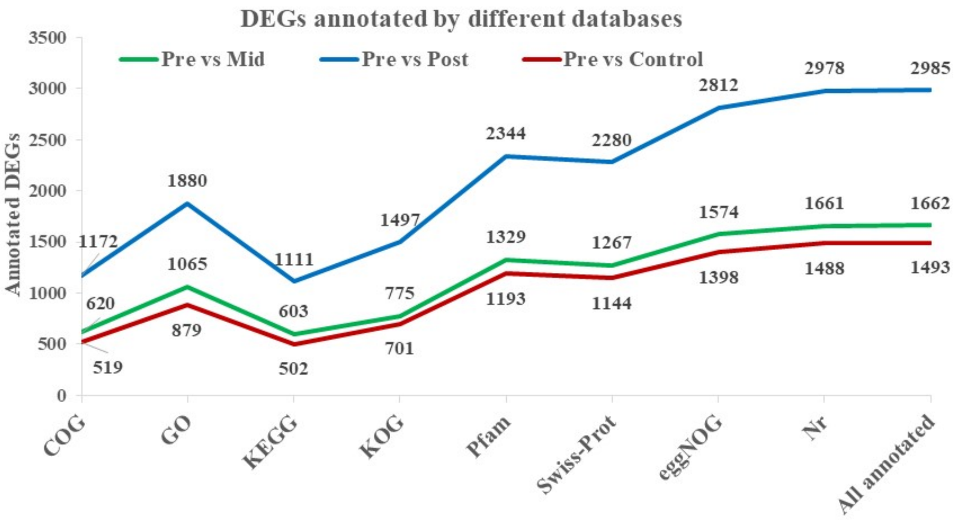

Figure S2. Functional annotations of DEGs against public databases.
